# Framing of female medical personnel during the COVID-19 pandemic: a case study of the Chinese official media

**DOI:** 10.1057/s41599-023-01749-0

**Published:** 2023-05-18

**Authors:** Cunling Gao, Hongfa Yi, Jinfu Wang, Shanshan Han

**Affiliations:** 1 School of Literature, Journalism and Communication, Qingdao University, Qingdao, China; 2 School of Journalism and Communication, Shanghai University, Shanghai, China; 3 School of Foreign Languages, Qingdao University, Qingdao, China; 4 School of Journalism and Communication, Hebei University, Baoding, China

**Keywords:** Cultural and media studies, Medical humanities, Health humanities

## Abstract

This paper analyzes the media frames adopted by the official WeChat and Sina Weibo accounts of the People’s Daily between January 1 and December 31, 2020, for reports about female medical personnel involved in pandemic prevention and control. Although the number of female medical personnel involved in pandemic prevention and control far exceeded that of their male counterparts, the extent of media reports on the former was far less than that of the latter. The human interest frame about female medical personnel was mainly applied, while the use of the action frame was less frequent, which highlighted the gender identity and family role of these women but weakened their professional identity. This was not conducive to praising the contributions of female medical personnel in fighting the pandemic. The media frames of reporting medical personnel in WeChat and Sina Weibo accounts of the People’s Daily are not always the same. After Wuhan’s lockdown ended on April 8, the proportion of the human interest frame of the report text of female medical personnel decreased, and the proportion of the action frame increased, while the proportion of the human interest frame of the report text of male medical personnel increased and the proportion of the action frame decreased. Previous studies mainly analyzed the use of the media frames of female news personalities, but few studies focused on whether women had the possibility of breaking away from the gender media frames. This study shows that some female medical personnel with exceptional professional competence are likely to transcend the gender media frames and receive similar coverage to that of male medical professionals, like Li Lanjuan and Chen Wei.

## Introduction

“One is not born, but rather becomes, woman” (De Beauvoir, 2009; p. 330). For this author, woman is the product of civilization construction that begins at the birth of a girl and continues throughout her whole life. In terms of gender relations, women become the second sex to men. The second sex means that the relationship between males and females is not a bipolar relationship like the north and south poles of a magnet, but a relationship between self and others in dualism. Women are not regarded as autonomous beings. Society defines women from the perspective of men.

In Chinese culture, women have long been in a subordinate position of being suppressed and enslaved. The male authority in feudal society of China constructed the moral standard of “three obediences and four virtues” to restrain women. Among them, “three obediences” means that a woman should obey her father before marriage, her husband after marriage, and her son after her husband’s death. “Three obediences and four virtues” also show the three main identities of women: as daughters, wives, and mothers. In the division of family roles, “men manage external affairs, women internal” was prioritized, which means that men were responsible for the external aspects of the family, while women were responsible for the family itself. This demanded that women be responsible only for weaving and sewing, cleaning and cooking, and caring for children at home, while outside the home was a public domain reserved merely for men.

In the last century, with social progress and the women’s liberation movement, the social status of Chinese women has improved. However, the inherent stereotype of gender still exists and can have an impact on women at different stages of life. For example, the idea of “son preference” continues to exist in some regions, and some regions have even formed an industrial chain for illegally identifying the sex of the fetus. According to a search on the China Judicial Document Network, nearly 500 cases of illegal identification of fetal sex have been prosecuted since 2010. For underage women, gender discrimination is widespread in education (Guo et al., 2022) and family life (Tian et al., 2018; Lin et al., 2021). For adult women, the workplace is an area where gender discrimination is concentrated. Research shows that there are gender stereotypes in job advertisements, and women have less chance of getting jobs in the job market than men (Woodhams et al., 2009; Zhang et al., 2021), while their income is far less than that of their male counterparts (Démurger et al., 2007; Chi and Li, 2014).

With the development of mass media, media have become a powerful factor in the construction and maintenance of gender notions. In some cases, mass media has even become a primary promoter of reinforcing inherent gender biases towards women. For instance, words like “Chinese Dama,” “female driver,” “leftover woman,” and others that involve women stereotypes stem from mass media constructions (Li, 2017; Feldshuh, 2018; Li and Luo, 2020). In addition, the researchers also found that the number of reports on female athletes in the Chinese media is relatively small, and the excessive attention to the gender role of female athletes is not conducive to highlighting their achievements in sports (Xu et al., 2018; Xu and Kreshel, 2021). Gender discrimination has brought negative impacts on Chinese society, including a decrease in population size, birth rates, working-age populations, accelerated aging, and exacerbation of the male marriage squeeze (Jiang et al., 2011). It is of great significance for women and the whole society to avoid the media continuing to report women in a way with gender stereotypes. In China, women make up the majority of professional groups like doctors and teachers, yet scholars have not shown sufficient concern regarding the media portrayal of Chinese working women groups. At the end of 2019, a large-scale outbreak of COVID-19 occurred in Wuhan, Hubei Province, China. A large number of reports involved female medical personnel. By analyzing the media reports of anti- pandemic female medical personnel, this paper explores the media presentation of Chinese working women groups and the possible media stereotypes.

## Literature review

### Media and gender discrimination

Feminists contend that gender inequality is the result of entire social and cultural constructions (Flax, 1987; Keller, 1988; De Beauvoir, 2009), this view contradicts to the essentialist view of gender. Recent studies have increasingly focused on the role of media in gender bias, sexism, and gender stereotypes. Scholars assert that mass media has become a pervasive and powerful factor in constructing and perpetuating inherent gender concepts (Busby and Leichty, 1993; Olson, 1994), which can construct, reflect, or reinforce gender stereotypes (Taylor, 2003; Lauzen et al., 2008; Holtzman and Sharpe, 2014: p. 46). Two different perspectives exist on the media’s representation of women: one view argues that women are underrepresented in news media (Kahn and Goldenberg, 1991; Matthews, 2007; Bloom, 2011; Conway and McInerney, 2012), while the other contends that some female groups are overrepresented by the media. Taking China as an example, Feldshuh (2018) analyzed unmarried women known as “leftover women” in Chinese media. The author believes that leftover women are not a demographic reality, but a result of media construction. Li and Luo (2020) analyzed the stigmatization of female drivers on Chinese social media. The author contends that too much discussion on traffic accidents involving female drivers on social media has created the myth of “bad women drivers.”

Research on the media framing of female news personalities has focused on the following groups: female political figures (e.g., female politicians and female political candidates), female criminals (e.g., female terrorist attackers), and female athletes. Studies have found that, relative to male political figures, the media concentrates more on aspects of female political figures’ appearances, family statuses, and daily lives, which lacks attention to their political orientations and policy views, thereby affecting the effectiveness of female political candidates’ campaigns and devaluing their political contributions (Aday and Devitt, 2001; Devitt, 2002; Garcia-Blanco and Wahl-Jorgensen, 2012; Ross and Comrie, 2012; Snipes and Mudde, 2020; Johnson-Myers, 2021). Moreover, Western media coverage of female political criminals, such as female terrorists, focuses disproportionately on personal information unrelated to criminal activity. These media use gender stereotypes to explain crimes and highlight the gender identity of female terrorist attackers rather than their criminal identity. This is not conducive to faithfully reflecting the realities of female terrorists involved in terrorist activities (Lavie-Dinur et al., 2015; Yarchi, 2014; La and Pickett, 2019; Auer et al., 2019; Shaban, 2020; Nacos, 2005). Through their analysis of major events like the Olympic Games, researchers found that the coverage of female athletes was significantly less than that of male athletes. In media coverage, female sports are portrayed as less important and less passionate than male ones. (Dashper, 2018; Angelini and Billings, 2010; Billings and Eastman, 2003).

### Frame and media frame

In Goffman’s view, “Framing is a process of giving meaning, interpretation, organization, and classification to experiences; these experiences shape cognitive perceptions and mediate in life situations” (Goffman, 1974: p. 18). Numerous scholars in the field of journalism and mass communication have reinterpreted framing and frame analysis based on Goffman’s view (Gitlin, 1980: p. 7; Entman, 1993; Pan and Kosicki, 1993; Semetko and Valkenburg, 2000; D’Angelo, 2002; Reese, 2007; Matthes and Kohring, 2008). Among them, Entman’s definition is the most cited. Entman (1993) argued that framing involves selecting some aspects of a perceived reality and making them more salient in a communication text in a way that promotes a particular problem definition, causal interpretation, moral evaluation, and/or treatment recommendation for the item described. Gitlin (1980: p. 7) argued that media frames are persistent patterns of cognition, interpretation, and presentation, involving selection, emphasis, and exclusion, by which symbol-handlers routinely organize discourse, whether verbal or visual.

With the development of social media, some scholars believe that it is necessary to study the media frame of new media (Nisbet, 2010; Morris and Ogan, 2018). Twitter is the new media platform that scholars pay the most attention to. A large number of studies of frame analysis for Twitter involves political issues, such as social protests (Bajpai and Jaiswal, 2011; Ashfaq et al., 2022; Cowart et al., 2022), party propaganda (Stier, 2016), election campaigns (Groshek and Al-Rawi, 2013), political events (Siapera et al., 2018; Šimunjak and Caliandro, 2020), etc. Health is another hot topic for frame analysis of Twitter (Pavlova and Berkers, 2022; Tahamtan et al., 2021). In addition, some scholars have conducted frame analysis on Facebook (Lin, 2016; Lev-On, 2019; Starr and Oxlad, 2021; Vu et al., 2021; Lee et al., 2022). Sina Weibo has become the Chinese version of Twitter. Zhao and Wang (2022) analyzed the Weibo media frame during the COVID-19 pandemic and found that the public challenged government media frames by creating new frames. Yang et al. (2019) found that People’s Daily, China Daily and the Chinese version of the Wall Street Journal’s coverage of the smog by their Weibo accounts has generated five frames.

### Two approaches of frame analysis

Researchers have divided media frames into Generic Frames and Issue-Specific Frames. The latter pertain to specific topics or news events, whereas generic ones are broadly applicable to a range of different news topics (De Vreese et al., 2001). Generic frames derive from the regularities characterizing the process of professional public communication per se (David and Baden, 2017: p. 8) and transcend thematic limitations, as they can be identified across different issues (Matthes, 2009). One researcher analyzed 15 major international journal media from 1990 to 2005 with frame-related studies and found that 78% of the papers adopted issue-specific frames and 22% applied generic frame. This research also identified 561 issue-specific frames and 29 generic frames (Matthes, 2009).

In frame analysis, researchers have taken two main approaches: inductive and deductive. The deductive approach begins with a specific set of frames, where researchers determine the definition and classification indicators of each frame before examining its occurrence in a given news story. The inductive approach, on the other hand, involves no predetermined frames and comes from systematic analysis and in-depth reading of the texts. Deductive approaches are mainly associated with generic frames, while inductive approaches chiefly involve issue-specific frames.

The inductive approach has become the most common research method in studies of women’s media frames. Researchers have used this approach to generalize different media frames (Nacos, 2005; Martini, 2018; Devitt, 2002; McManus, 2013). Generic frames appear more frequently in news stories compared to other types of texts. Scholars have summarized some generic frames based on different criteria, laying the foundation for the adoption of deductive methods to study media frames. Semetko and Valkenburg (2000) outlined five common generic frames: conflict frame, human interest frame, economic consequences frame, morality frame and responsibility frame. The above generic frames have significantly influenced a large number of scholars to employ deductive methods for frame analysis (Luisi et al., 2018; Schulenberg and Chenier, 2014; Igartua et al., 2005; Dimitrova et al., 2005).

This paper focuses on the official WeChat and Sina Weibo accounts of People’s Daily. WeChat is China’s leading messaging and social media application with 1.2 billion monthly active users. The Official Account is one of its functions, which allows authors to publish text, pictures, and videos that can be shared by other users on their personal spaces. Sina Weibo is China’s most popular social media platform, similar to Twitter, with 582 million monthly active users. This paper uses a deductive approach and a set of generic frames to analyze the media frames of female medical personnel. The study aims to answer the following questions: What frames were presented by the WeChat and Sina Weibo accounts of People’s Daily in their coverage of female medical personnel fighting against the pandemic? How do the media frames of female medical personnel differ from those of male medical personnel? What kind of effects do the media frames of female medical personnel bring, and what kind of female media image do they present? Is it possible for women in news media to transcend the inherent gender media frame?

## Research methodology

This study used “Novel Coronavirus”, “Pandemic” and Chinese abbreviation for “Novel Coronavirus” (i.e., Xin Guan) as keywords, and applied crawler software to obtain 2,460 WeChat articles and 6,044 Sina Weibo posts of People’s Daily from January 1 to December 31 2020. People’s Daily is the organ of the Communist Party of China and the most authoritative official media in China. In many cases, it can represent the voice and will of the Chinese government. The paper version of the People’s Daily is mainly distributed by government agencies, and its new media reach is even greater. The paper version of People’s Daily is limited by space, its reports on social media are richer and more time-sensitive. People’s Daily has been a typical representative of media integration in China, and its exploration of this field has exemplary significance. The WeChat Public Account of People’s Daily has over 20 million followers, ranking first among Chinese media, and the official Sina Weibo account of People’s Daily has nearly 150 million followers. In addition, the new media of People’s Daily performed outstandingly in the pandemic coverage. During the Wuhan pandemic, the official WeChat and Sina Weibo accounts of People’s Daily released a series of news and achieved good communication effects. According to the information disclosed in March 2020, the average daily readings of People’s Daily Sina Weibo account since the pandemic coverage exceeded 1 billion times, and the WeChat Public Account published more than 20 related reports every day, with an average daily reading of more than 30 million times. This is the main reason why this paper chooses it as the research object.

This researcher manually reviewed all the crawled Sina Weibo posts and WeChat articles before selecting those involving medical personnel as the study sample. The medical personnel referred to in this paper include doctors, nurses, and disease control experts involved in the prevention and control of the pandemic. In terms of gender identification, the researchers relied on the third person “she” and “he” or the relationship “mother,” “father,” “wife,” and “husband” in the texts and identified physical characteristics with the help of news pictures. The third way was to search for the names of relevant medical personnel and confirm them based on public information on the Internet. Medical personnel whose gender could not be identified by the above three means were not included in the study sample. WeChat articles or Sina Weibo posts involving both male and female medical personnel were judged according to their subject matter or contents to determine which gender was specifically preferred. After screening, a total of 54 Sina Weibo posts mainly reporting female medical personnel and 210 posts reporting male medical personnel were retrieved, while 49 WeChat articles mainly reporting female medical personnel and 103 articles for male medical personnel were respectively included in the study.

The total number of WeChat articles and Sina Weibo posts mainly reporting on female medical personnel was 103, while that reporting on male medical personnel was 313, which is three times more. According to data released by the National Health Commission of China on March 8, 2020, 346 medical teams had arrived in Hubei, with a total of 42,600 people. Among them, 28,000 were female, accounting for 65.7% (Li, 2020), and they also comprised the majority of the medical personnel in the national fight against the pandemic. However, female medical personnel did not receive the corresponding coverage scale. This was not conducive to highlighting their contributions to the fight against the pandemic.

This study takes a deductive approach to analyze the media frames of all 416 Sina Weibo posts and WeChat articles mentioned above. The researcher fine-tuned Semetko and Valkenburg’s five generic frames to make them more consistent with the research theme of this paper. Besides that, Shih et al. (2008) analyzed the media frame of public health infectious diseases by proposing an action frame. Action frame involves any measures and actions to address the diseases, such as prevention and treatment, etc. This frame was further applied in the analysis of European media coverage of H1N1 influenza (Rossmann et al., 2018). In this paper, the conflict frame, human interest frame, morality frame, responsibility frame, and action frame were all analyzed deductively, using on Semetko and Valkenburg’s classification indicators and refining each frame into several independent questions. The economic consequence frame was not used because news reports about medical workers responding to the pandemic were not economically relevant. These indicators were used to code the screened WeChat articles and Sina Weibo posts to identify the most dominant frames of each particular blog post. Two coders coded the frames independently, and the coding reliability was set to *Scott’s pi* = 0.87. The specific frame identification indicators are as follows (Table 1):

**Table 1:** Frame and secondary indicators.

Frame	Frame content	Secondary indicators
Conflict frame (Semetko and Valkenburg, 2000)	Which emphasizes conflicts between individuals, groups, or institutions as a tool of capturing audience interests.	(1) Whether inconsistencies, disagreements, or oppositions in views, actions, or goals are involved.
		(2) Whether the individual is placed in conflicting or dilemma situations.
		(3) Whether it involves competition between individuals.
Human interest frame (Semetko and Valkenburg, 2000)	Which brings a human face or an emotional angle to the presentation of an event, issue, or problem.	(1) Whether it contains details, scenes, pictures or videos that can trigger emotions like happiness, anger, sympathy, sadness, fear and disgust in the audience.
		(2) Whether or not the character is shown in a personal life role, identity, or manner of behaviors not related to his or her professional identity.
		(3) Whether to highlight the emotions, feelings, and other aspects of the characters as individual human beings.
Morality frame (Semetko and Valkenburg, 2000)	Which puts the event, problem, or issue in the context of religious tenets or moral prescriptions.	(1) Whether there is a content that judges the moral character of the person concerned.
		(2) Whether to highlight the facts that reflect the spiritual qualities of the person concerned.
		(3) Whether certain types of social and ethical codes are mentioned in the report.
Responsibility frame (Semetko and Valkenburg, 2000)	Which presents an issue or problem in order to attribute responsibility for its cause or solution to either the government or an individual or group.	(1) Whether to specify the behaviors of some individuals, organizations or groups that lead to some negative consequences.
		(2) Whether certain individuals, organizations, or groups are identified as bearing responsibility for solving a problem.
Action frame (Shih et al., 2008)	Which involves any measures and actions to address the diseases, such as prevention and treatment, etc.	(1) Whether it involves an initiative taken by the person for responding to an issue (pandemic prevention and control, etc.).
		(2) Whether or not it involves a person to publicly express opinions, views on a certain issue (pandemic prevention and control, etc.).

## Research findings

### Frame usage

The study found that the People’s Daily WeChat and Sina Weibo accounts primarily used the human interest frame and action frame in their news coverage of the Novel Coronavirus outbreak involving medical personnel. The responsibility frame, morality frame, and conflict frame were less commonly applied. The human interest frame mainly covered the family relationships and emotions of medical personnel, such as the Sina Weibo post on February 3, “White nurse and firefighter’s wedding,” and another on April 19, “Forward your blessings! A nurse and her police boyfriend got married.” The WeChat article on February 25 entitled “When mom stayed on the front line of the fight against the pandemic, the 6-year-old brother was so strong that he was able to console his brother missing his mother,” also falls under this frame. In addition, the human interest frame also reflected the hard work and dedication of medical staff through depictions of their body parts, such as the WeChat article post on February 1 entitled “Heartache! This is the hand of a 22-year-old nurse,” and another on March 8 entitled “Beauty can’t hide! Comparison pictures of female nurses before and after anti-pandemic fight.” The Sina Weibo post “Traces of a mask on the face of Li Lanjuan” on February 20 also falls under this frame. Li Lanjuan is a female academician of the Chinese Academy of Engineering, an expert in infectious diseases, and a member of the high-level expert group of the National Health Commission whose words and deeds gained widespread media attention during the outbreak of the Novel Coronavirus.

The action frame primarily focused on pandemic diagnosis, protection knowledge popularization, patient treatment, and other practical actions to combat the pandemic, such as the June 29 Sina Weibo post “Wu Zunyou said between 10 and 20 million infected persons will be diagnosed worldwide in a much shorter period of time,” the June 20 Sina Weibo post “Experts: Beijing pandemic prevention cannot copy the traditional Chinese medicine prescription used in Wuhan,” and the February 3 Sina Weibo post “Zhong Nanshan’s team laboratory isolated live virus in patient’s feces,” and the March 2 WeChat article entitled “The lung is not existent anymore, the first case of Novel Coronavirus autopsy report released”, etc. Wu Zunyou and Zhong Nanshan were both male medical experts who received widespread media and public attention during the COVID-19 outbreak. Wu Zunyou is the chief epidemiologist of the Chinese Center for Disease Control and Prevention (CDC), who often participated in official press conferences during the Novel Coronavirus outbreak. Zhong Nanshan is an academician of the Chinese Academy of Engineering and the head of the Senior Expert Group of the National Health Planning Commission, and he made important contributions to the SARS outbreak in 2003 and the Novel Coronavirus outbreak in 2019.

On April 8, 2020, after 76 days of closure, Wuhan lifted its control measures on outbound traffic and resumed external traffic in an orderly manner. Taking April 8 as the boundary, reports on medical staff fighting the pandemic in People’s Daily’s WeChat and Sina Weibo were divided into two stages. The first stage was from January 1, 2020, to April 8, 2020, and the second stage was from April 9, 2020, to December 31, 2020. It was found that there were significant differences in the choice and use of frames between the two stages of news reporting (see Tables 2 and 3). Compared to the previous period, the proportion of reports mainly involving male medical personnel with action frames in the latter period decreased significantly (WeChat: from 64.7% to 44.2%; Weibo: from 74.4% to 70.5%; Weibo and WeChat total: from 71.1% to 62.1%), while the proportion of reports mainly involving female medical personnel with action frames increased significantly (WeChat: from 16.1% to 22.2%; Weibo: from 18.9% to 41.2%; Weibo and WeChat total: from 17.6% to 31.4%). The proportion of reports mainly involving female medical personnel with human interest frames also decreased significantly (WeChat: from 80.6% to 77.7%; Weibo: from 67.5% to 41.2%; WeChat and Weibo total: from 73.5% to 60.0%). The human interest frames of reports mainly involving male medical personnel did not change significantly (WeChat: from 35.2% to 48.0%; Weibo: from 16.3% to 12.5%; WeChat and Weibo total: from 22.8% to 23.7%). Therefore, the news frames used by People’s Daily when reporting on medical staff against the pandemic are not invariable, but changes with the reporting stage and the pandemic prevention and control situation.

**Table 2:** Frame comparison and chi-square test (frequency/proportion) (Jan.1 to Apr.8, 2020).

Frame (1.1–4.8)	Sina Weibo		WeChat	
	Mainly involving female	Mainly involving male	Mainly involving female	Mainly involving male
Conflict frame	1 (2.7)	0 (1.0)	1 (3.2)	0 (0.0)
Human interest frame	25 (67.5)	16 (16.3)	25 (80.6)	18 (35.2)
Morality frame	3 (8.1)	6 (6.1)	0 (0.0)	0 (0.0)
Responsibility frame	1 (2.7)	3 (3.0)	0 (0.0)	0 (0.0)
Action frame	7 (18.9)	73 (74.4)	5 (16.1)	33 (64.7)
Total	37 (100.0)	98 (100.0)	31 (100.0)	51 (100.0)
*χ*^2^	40.037***		18.807***	

***p < 0.001.

**Table 3:** Frame comparison and chi-square test (frequency/proportion) (Apr.9 to Dec.31, 2020).

Frame (4.9–12.31)	Sina Weibo		WeChat	
	Mainly involving female	Mainly involving male	Mainly involving female	Mainly involving male
Conflict frame	0 (0.0)	2 (1.7)	0 (0.0)	0 (0.0)
Human interest frame	7 (41.2)	14 (12.5)	14 (77.7)	25 (48.0)
Morality frame	1 (5.8)	9 (8.0)	0 (0.0)	1 (1.9)
Responsibility frame	2 (11.7)	8 (7.1)	0 (0.0)	3 (5.7)
Action frame	7 (41.2)	79 (70.5)	4 (22.2)	23 (44.2)
Total	17 (100.0)	112 (100.0)	18 (100.0)	52 (100.0)
*χ*^2^	10.163*		5.181	

^*^p < 0.05.

### Framing effect

The selection and application of media frames exerted a “framing effect” on society and audiences. Experimental studies have shown that frames draw attention to particular aspects of the reality described, which logically means that they simultaneously direct attention away from other aspects (Entman, 1993). As shown in Table 4, the *χ*^2^ test found a significant difference in media frames by gender ($\chi ^2 = 52.055,\,p \,<\, 0.001;\,\chi ^2 = 22.884,\,p \,<\, 0.001$). Moreover, the proportion of human interest frames used in reporting on female medical personnel (59.2% and 79.6%) was much higher than that used in reporting on male medical personnel (14.2% and 41.7%), while the proportion of action frames used in reporting on female medical personnel (25.9% and 18.3%) was much lower than that used in reporting on male medical personnel (72.3% and 54.3%) in People’s Daily’s Sina Weibo posts and WeChat articles. The human interest frame was mainly related to two aspects: the role of women in the private sphere such as family and marriage, and the portrayal of women’s body parts.

**Table 4:** Frame comparison and chi-square test (frequency/proportion) (Jan.1 to Dec.31, 2020).

Frame (1.1–12.31)	Sina Weibo		WeChat	
	Mainly involving female	Mainly involving male	Mainly involving female	Mainly involving male
Conflict frame	1 (1.8)	2 (0.9)	1 (2.0)	0 (0.0)
Human interest frame	32 (52.9)	30 (14.2)	39 (79.6)	43 (41.7)
Morality frame	4 (7.4)	15 (7.1)	0 (0.0)	1 (0.9)
Responsibility frame	3 (5.5)	11 (5.2)	0 (0.0)	3 (2.9)
Action frame	14 (25.9)	152 (72.3)	9 (18.3)	56 (54.3)
Total	54 (100.0)	210 (100.0)	49 (100.0)	103 (100.0)
*χ*^2^	52.055***		22.884***	

***p < 0.001.

There is a significant amount of content in WeChat articles and Weibo posts that highlight the roles of female medical personnel as wives, lovers, and mothers. For example, on February 3, there was a Weibo post “The wedding in a long distance for a nurse in white clothes and a fireman”, and on April 29, there was a WeChat article entitled “The nurse in Hubei who once called out her daughter to do her homework was seriously suffering from cancer. Netizens wish her to be better!”. In contrast, there are very few reports highlighting the role of male medical personnel in family and marriage. In all the reports analyzed, “husband,” “father,” “son,” and other identities representing family relationships rarely appear. Whereas cropped hair, indentations on the face, and burned hands may seem to praise women’s dedication, they actually imply a deeper connotation of morally judging the body as an esthetic subject. This influences the reader’s interpretation of the message, with selfless devotion replacing professional skills as the major criterion for measuring female medical personnel fighting the pandemic, and selfless moral models replacing skilled professionals as their primary image. The Feb. 12 Sina Weibo post “Super valiant! Female medical personnel shaved off the hairs at the back of their heads,” to which netizen @fanziyang commented: “For the sake of convenience in work, they have become our female knight-errant instead!” The term “knight-errant” is more of an ethical evaluation of female medical personnel. Furthermore, the reinforcement of the human interest frame and the weakening of the action frame have obscured the professional competencies of female health workers and downplayed their actual and professional actions in the fight against the pandemic. These media frames are influenced by established gender perceptions and further reinforce these stereotypes of both genders.

### Transcending gender frames

The study found that Chinese official media displayed gender bias in reporting on female medical personnel. By using different media frames, the news media presents the image of female medical personnel differently from that of men, and the inherent gender cognition of society permeates into the news reports of the media. Given this background, is it possible for women to transcend the gender bias of the media? Beauvoir (2009: p. 813) pointed out, “It is through work that woman has been able, to a large extent, to close the gap separating her from the male.” However, this study shows that the majority of female medical personnel did not really bridge the gap with their male counterparts. In the media reports, they still appeared in a different image from men. Nonetheless, this study also found that the gender media frame did not target all female medical personnel, and the media adopted media frames similar to that of male medical personnel when reporting on some female medical personnel and presented a similar image to their male counterparts.

Taking Li Lanjuan as an example. Li Lanjuan’s name appeared in the title of 4 Wechat articles, among which 3 articles used action frame (75%) and 1 article used human interest frame (25%). When reporting on Li Lanjuan, the WeChat official account of People’s Daily used the action frame more frequently than the average male medical staff (54.3%), and used the human interest frame less frequently than the average male medical personnel (41.7%). Li Lanjuan appeared 8 times in Weibo posts mainly involving female medical personnel, among which 5 posts used the action frame (62.5%) and 3 posts used the human interest frame (37.5%). Although the frequency of action frame involving Li Lanjuan was lower than the average of male medical personnel (72.3%), it was much higher than the average of female medical personnel (25.9%). The probability of using the human interest frame when reporting on Li Lanjuan by Weibo was higher than the average of male medical personnel (14.2%), but much lower than the average of female medical personnel (59.2%).

Let’s compare Li Lanjuan’s reporting frame with Zhong Nanshan’s. Zhong’s name appeared in the title of 27 WeChat articles, of which 20 used the action frame (74%) and 5 used the human interest frame (18.5 %). In other words, the probability of using the action frame when reporting Li Lanjuan by People’s Daily WeChat was equal to that of Zhong Nanshan’s, while the probability of using the human interest frame was slightly higher than Zhong Nanshan’s.

The above content mainly emphasizes the actual actions that Li Lanjuan took to fight the pandemic, such as the Sina Weibo posts “Li Lanjuan claims that it is not correct that the Corona Virus exists for 20 years in minus 20 °C,” “Li Lanjuan leaving Wuhan while warning of two great dangers,” and the WeChat articles entitled “When can Wuhan be unsealed? Academician Li Lanjuan said like this,” “Li Lanjuan: after ‘zero increase’ in Wuhan’s pandemic, two issues still need to be highly emphasized.” Chen Wei is an academician of the Chinese Academy of Engineering and has made contributions to the research and development of COVID-19 vaccines in China. When people’s Daily reported on Chen Wei on WeChat and Sina Weibo, they all paid attention to the progress and achievements of her vaccine experiments.

In our view, the action frame is more conducive to demonstrating the professional identity of medical personnel and their contribution to the fight against the pandemic, while the human interest frame highlights the personal side of medical personnel. In media reports, Li Lanjuan and Chen Wei are fighting the pandemic with their sublime expertize, researching and developing COVID-19 vaccines and expressing their opinions, predictions, and warnings on the pandemic. They demonstrate their image as female scientists with conspicuous professionalism and great contributions to the fight against the pandemic, which is in line with their male counterparts like Wu Zunyou and Zhong Nanshan. Therefore, the gender media frame is not inevitable in news reports, and it is possible for women to be liberated from the gender media frame.

## Discussion

Although the quantity of female medical personnel involved in the fight against the COVID-19 pandemic in China far exceeded that of men, the number of reports on them on the official WeChat and Sina Weibo accounts of People’s Daily was much lower than those on men. The frequency of action frame in the reports of female medical personnel was much lower than that of the male ones. And the frequency of human interest frame in the reports of female medical personnel was much higher than that of male ones.

This study focuses on the media frames used by Chinese official media to report on female medical personnel during the COVID-19 pandemic. There have been relatively few studies on the media frames of female medical personnel in the fields of humanities and social sciences, journalism, and communication. This study used a deductive approach in frame analysis, employing a set of generic frames to examine relevant coverage texts, which is another innovation of this study. The finding that female medical personnel did not receive the number of reports they deserved is consistent with previous studies (Kahn and Goldenberg, 1991; Matthews, 2007; Bloom, 2011; Conway and McInerney, 2012). Previous researchers have argued that the media frames used by news outlets to report on female news personalities were detrimental to the effective recognition of female news personalities (Johnson-Myers, 2021; La and Pickett, 2019). However, they did not answer the question of whether female news personalities can transcend the inherent gender media frame. The innovative discovery of this study is that female news personalities have the possibility of transcending the inherent gender media frame, but that only exists in outstanding female news personalities. In the reports on the WeChat and Sina Weibo accounts of People’s Daily, Li Lanjuan and Chen Wei demonstrate their image as scientists with conspicuous professionalism and great contributions to the fight against the pandemic, which is in line with their male counterparts like Wu Zunyou and Zhong Nanshan. In the case of this research, women liberate from the gender media frame due to their sublime expertize and supreme professional influence, even going beyond men. Specifically, the research and development of vaccines, virus analysis, and other work carried out by Li Lanjuan and Chen Wei have strong news value and are suitable for reporting as dynamic news. This undoubtedly increases the proportion of the action frame in the reports of Li Lanjuan. Additionally, as well-known experts, Li Lanjuan and Chen Wei have their own traffic value in the network communication environment, and their names can attract netizens’ attention when they appear in the media. However, ordinary female medical personnel can only attract users’ attention with sentimental content full of human feelings, which increases the frequency of the emergence of human interest frames.

The ordinary women are difficult to cast off the gender stereotypes that gender media frame has produced. Female medical personnel were often portrayed in the news primarily as wives and mothers in their private roles, rather than as medical professionals. This led to the selfless moral models rather than skilled professionals being the dominant media image of female health workers, which was not conducive to highlighting the contributions of female health workers in the fight against the pandemic.

This discovery indicates that gender stereotypes in the field of Chinese news reporting may continue to improve in the future. With changes in the social environment, Chinese women have gained increasing independence and self-sufficiency, shedding their dependence on men. Female students and staff are excelling in universities and workplaces, and the rise of women has become an important social trend. The trend of being single, delaying childbirth, and having fewer children among Chinese women allows them to focus more on things outside of their families, which helps them achieve more outstanding performance in the public sphere. This means that more and more Chinese women are likely to become outstanding female news personalities and transcend the inherent gender media frame.

At the same time, the media frame of medical personnel on the official WeChat and Weibo accounts of People’s Daily is not static and exhibits obvious phased characteristics. Compared to reports before April 8, 2020, which mainly involved female medical personnel, there was a significant increase in the proportion of action frames and a decrease in the proportion of human interest frames on April 9 and after. Before April 8, the COVID-19 pandemic had posed a significant challenge to the Chinese government’s public health emergency response capacity. As the official media, People’s Daily needed to provide positive guidance to offset the negative anti-pandemic information from Wuhan and guide public opinion. Some humane performances of female medical personnel in the fight against the pandemic became effective materials for this purpose. The end of the lockdown of Wuhan on April 8 means that China has made significant progress in combating the COVID-19. Preventing the recurrence of the pandemic and dispelling the public’s fear of the pandemic became the main tasks. In order to achieve this goal, the official WeChat and Weibo accounts of People’s Daily began to highlight assertions, reminders, and opinions of authoritative figures in reports on female medical personnel.

The media frames used to report on female medical personnel during the COVID-19 pandemic did not originate from the news media itself, but from a complex social and cultural background. In Chinese cultural tradition, patriarchal oppression of women is the fundamental reason for the formation of gender media frames. In patriarchal discourse, women are viewed as men’s appendages, whose value lies in selfless devotion and self-sacrifice for men. Repeated reports of female medical personnel with traces of mask and short haircuts may seem to praise their selflessness, but its essence is the tradition of the sacrifice of women under the patriarchy. In addition, women are alienated as maids of the household under the patriarchy. The media’s focus on women’s roles as mothers, wives, and else in the above reports is a manifestation of alienation of women under the patriarchal system.

The suffering experienced by female medical personnel in the media is not an isolated occurrence. Chinese media tend to have varying degrees of gender bias when reporting on other female news personalities. Today, with the continuous advancement of the medialization process, how to report women in the media has become a significant proposition related to social development and human progress. How to get rid of the gender stereotypes brought by the gender media frame in news reports has become a topic to be further studied. The main limitation of this study is that only one media, People’s Daily, is selected, and the amount of data is relatively small. Additionally, this study only analyses the media’s report texts and does not include interviews or questionnaires to investigate the actual impact of the media reports on the public.

## Conclusion

This study demonstrates that the official WeChat and Sina Weibo accounts of the People’s Daily have a clear gender bias when reporting on female medical personnel fighting against the epidemic, mainly manifested in the number of reports and media frames. Even though 2/3 of the medical teams going to Hubei to fight the epidemic were women, however, statistics from January 1 to December 31, 2020, show that the official WeChat and Sina Weibo accounts of the People’s Daily reported only 1/3 of the number of female medical staff as the male medical staff. They used a higher proportion of human interest frame and a lower proportion of action frame than male medical staff when reporting on the female medical staff. At the same time, this study also found significant changes in the media frame used for different reporting stages and targeting different female news figures. After the lifting of lockdown in Wuhan on April 8, 2020, there was a shift in the nature of reports on the People’s Daily official WeChat and Sina Weibo accounts. The proportion of reports featuring male medical staff using an action frame decreased, while the proportion of reports using a human interest frame increased. Conversely, the proportion of reports featuring female medical staff using an action frame increased, and the proportion of reports using a human interest frame decreased. When they reported on female medical staff with exceptional professional competence, the proportion of action frame was higher than that ordinary female medical staff, and the proportion of human interest frame was lower than that of ordinary female medical staff. Although women with outstanding professional ability can surpass the gender media frame, gender prejudice in news reports, in general, is not conducive to showing the contribution of women’s medical staff against the epidemic. Future research should examine more conditions under which more categories of women are able to transcend the gender frames. In addition, the effects of transcending the gender frames may be the focus of follow-up research.

## Data Availability

The datasets generated during and/or analyzed during the current study are available from the corresponding author on reasonable request.
